# Dengue algorithms integrated into the IMCI guidelines: An updated assessment in five Southeast-Asian countries

**DOI:** 10.1371/journal.pntd.0010832

**Published:** 2022-10-11

**Authors:** Stephanie Petzold, Kerstin D. Rosenberger, Bridget Wills, Jacqueline Deen, Martin W. Weber, Thomas Jaenisch, IDAMS IMCI study group

**Affiliations:** 1 Heidelberg Institute of Global Health, Heidelberg University Hospital, Heidelberg, Germany; 2 Section Clinical Tropical Medicine, Heidelberg University Hospital, Heidelberg, Germany; 3 Institute of Medical Statistics and Computational Biology, Faculty of Medicine and University Hospital Cologne, University of Cologne, Cologne, Germany; 4 Oxford University Clinical Research Unit, Wellcome Trust Asia Programme, Ho Chi Minh City, Vietnam; 5 Centre for Tropical Medicine and Global Health, Nuffield Department of Medicine, University of Oxford, Oxford, United Kingdom; 6 Institute of Child Health and Human Development, National Institutes of Health, University of the Philippines, Manila, Philippines; 7 WHO Regional Office for Europe, Office for quality of care, Athens, Greece; 8 Center for Global Health, Colorado School of Public Health, Aurora, Colorado, United States of America; McGill University Faculty of Medicine and Health Sciences, CANADA

## Abstract

**Background:**

Dengue is not included explicitly in the WHO Integrated Management of Childhood Illness (IMCI) algorithm. However, the assessment, classification and management of dengue has been incorporated into several IMCI country adaptations. We aimed to evaluate the dengue algorithms incorporated into IMCI guidelines and discuss the need for harmonization, including an extension of the age range for IMCI.

**Methods:**

This study included three steps. First, we investigated dengue algorithms incorporated into five Southeast-Asian (Myanmar, Philippines, Vietnam, Indonesia, Cambodia) country IMCI guidelines through a desk-based analysis. Second, we conducted an expert survey to elicit opinions regarding the integration of dengue and extension of the age range in IMCI. Third, we compared our findings with data from a large multicentric prospective study on acute febrile illness.

**Results:**

We found considerable heterogeneity between the country specific IMCI guidelines in the dengue algorithms as well as classification schemes. Most guidelines did not differentiate between diagnostic algorithms for the detection of dengue versus other febrile illness, and warning signs for progression to severe dengue. Our expert survey resulted in a consensus to further integrate dengue in IMCI and extend the age range for IMCI guidelines beyond 5 years of age. Most of the interviewees responded that their country had a stand-alone clinical guideline for dengue, which was not integrated into the IMCI approach and considered laboratory testing for dengue necessary on day three of consecutive fever. Using data from a large multicentric study of children 5–15 years of age, we could confirm that the likelihood of dengue increased with consecutive fever days. However, a significant proportion of children (36%) would be missed if laboratory testing was only offered on the third consecutive day of fever.

**Conclusions:**

This study supports the extension of the IMCI age range beyond 5 years of age as well as the inclusion of dengue relevant content in the algorithm. Because of the challenge of distinguishing dengue from other febrile illnesses, simple laboratory testing (e.g., full blood count) should be offered at an early stage during the course of the illness. Testing only children with consecutive fever over 3 days may lead to an underdiagnosis of dengue among those with acute febrile illness in children 5–15 years of age. In addition, specific laboratory testing for dengue should be made available to peripheral health facilities.

## Author summary

The Integrated Management of Childhood Illness (IMCI) was developed for the identification and treatment of major childhood illnesses based on a syndromic approach that can be applied by local health care workers in rural health facilities. Dengue represents an important differential diagnosis for children and adolescents with acute febrile illness in Southeast Asia but is not officially included in the fever module of IMCI. Local adaptations in different countries, however, have integrated dengue into their guidelines. We analysed the available local IMCI guidelines in Southeast Asia, conducted an expert survey, and investigated out findings using data from a large multicentric prospective study on acute febrile illness. Our findings support the extension of the age range for IMCI and the inclusion of dengue relevant content. When children presented with fever, the likelihood of dengue increased with consecutive fever days. We were able to quantify how likely dengue is as a cause of fever in children living in endemic areas, stratified by duration of fever. This evidence is useful for policy makers to consider the effect of more specific diagnostic tools for the monitoring of early dengue.

## Introduction

An estimated 390 million dengue virus infections occur annually across the globe [1], with Asia bearing around 70% of the global burden [2]. In South and Southeast-Asian countries dengue causes significant childhood morbidity and mortality [3]. During the COVID-19 pandemic, dengue cases even increased in many countries as for example Thailand, Ecuador, and Brazil [4]. Clinical manifestations of dengue range from mild symptoms to severe life-threatening disease [1,2,4]. Although the underlying pathophysiology of severe dengue is not completely understood, the risk is increased during secondary dengue infections [5–8]. Furthermore, a shift towards older age groups was observed in some countries (e.g., Thailand) in the last decades, highlighting the importance of adolescents in the clinical epidemiology [9,10].

Since the early manifestations of dengue are non-specific, there is considerable overlap between dengue and other acute febrile illnesses, which poses a major diagnostic challenge for clinicians, especially when confirmatory testing is not available [11]. Despite efforts to establish a validated screening tool based on clinical and simple laboratory parameters for the early detection of dengue, no such tool has been widely implemented [12,13]. Aside from clinically distinguishing dengue from other acute febrile illnesses, clinicians are also face with the challenge of correctly identifying dengue patients who are likely to progress to a more severe course of disease and who potentially need to be monitored more closely or referred [14].

The Integrated Management of Childhood Illness (IMCI) is a strategy developed by the World Health Organization (WHO) and the United Nations Children Fund (UNICEF) for the identification of childhood illnesses, treatment of major illnesses and referral of severely ill children, as well as the reduction of preventable childhood mortality [15]. Flow-chart diagrams provide algorithms to be followed by health personnel in primary care settings for the assessment, classification and treatment of illnesses using a syndromic approach [16]. The IMCI chart booklet is divided into two main parts, one of which focuses on sick children aged between 2 months and 5 years and the other on sick young infants aged up to 2 months, because clinical signs and case management procedures differ between these age groups. After the assessment and classification of the illness, the IMCI algorithms follow a flowchart using a colour-coded scheme: green for mild illness, yellow for moderate illness (with a daily follow-up and advice on when to return), and red for severe illness (requiring urgent referral to hospital). For example, a child with any general danger sign (e.g., inability to drink or breastfeed, vomiting) needs urgent attention and referral. If there is no general danger sign, the health worker asks whether the child has a cough or difficult breathing, diarrhoea, fever, or ear problems, each of which has a separate module. The IMCI strategy has been shown to reduce mortality and disability in under 5-year-olds [17]. Currently, dengue is not included in the generic WHO IMCI algorithm. The implementation of a generic IMCI strategy requires, besides coordination among health programs on different national and international levels, differentiated and harmonized epidemiological data from each country. Currently, dengue-specific content has been incorporated into some Asian IMCI adaptations for children aged 2 months to 5 years. However, most of these adaptations have not been tested for their effectiveness [18,19]. The aim of the study was to compare current Southeast-Asian dengue guidelines and IMCI algorithms, to and discuss the need for an extension of the age range as well as the adaptation of dengue-specific content. More specifically, we focused on the signs and symptoms for dengue vs. other febrile illness as well as the pending decisions regarding timing of laboratory investigations or hospitalization.

We used data from the EC-funded IDAMS study (www.idams.eu). Ethical approval has been described in detail in the study protocol manuscript [20]. In brief, the research project followed national and international ethics standards with ethical approval obtained from responsible international and national boards for each participating country.

## Methods

The methodology is comprised of three components. The first is a desk-based analysis of dengue algorithms incorporated into IMCI guidelines from Southeast-Asian countries. The second is a survey of experts (clinicians and researchers) from the region to elicit their opinion regarding the IMCI documents, specifically regarding the extension of the age-range of IMCI and the optimal point in time for standard laboratory testing (complete blood count including platelet and white blood cell count)–which in a third step was validated with data from a large multicentric prospective study acute febrile illness study [20].

### Desk-based analysis of dengue algorithms incorporated into IMCI guidelines

IMCI clinical guidelines were collected from local sources in Southeast Asian countries following an expert meeting in 2015 [21], which was part of the International Research Consortium on Dengue Risk Assessment, Management and Surveillance (IDAMS) [20] project, and updated in 2021. Data were extracted to obtain the signs and symptoms used for the assessment and classification of dengue and for referral to hospital. We compared the Southeast-Asian dengue IMCI algorithms to the WHO 2009 dengue guidelines [22], which were based on the results of a multicentre prospective study in four Southeast Asian and three Latin American countries [23]. The WHO 2009 dengue guidelines classify the illness into three categories according to clinical severity, which parallel the colour coded IMCI system of severity.

### Survey of experts

We conducted a survey of experts’ opinions in June and July 2020 regarding the integration of dengue recommendations into the IMCI algorithm, by using a standard questionnaire (S1 Appendix). The experts were attendees of a 2015 expert meeting [21], and included clinical and public health experts from the Southeast-Asian region. The survey focused on clinical aspects, indications for basic laboratory testing (full blood count including platelet count), and guideline standards. In addition, we included technical aspects such as age extension of IMCI and the integration of dengue into the fever section of the IMCI algorithm.

### Assessment of optimal timing for dengue laboratory testing

In order to establish a standard recommendation for the timing of laboratory testing (e.g., full blood count including platelet count and hematocrit) during an acute febrile illness in a dengue-endemic area, we compared the experts’ opinion on dengue laboratory testing with the results from the IDAMS study. For the comparative analysis, we included a sub-cohort of Asian children (5–15 years of age) and assessed fever duration (consecutive fever days) with the likelihood of a positive or negative dengue test result. All children either had documented or history of fever within the last 24h at enrolment. Clinical and vital signs as well as dengue-specific symptoms were recorded daily. Dengue was confirmed by polymerase chain reaction (PCR) or NS1 rapid diagnostic test. We evaluated (1) the likelihood of dengue according to the number of consecutive days of fever, and (2) how many children would be missed if testing is conducted after a certain number of consecutive days of fever and. Fever days were defined as days with measured fever (above 37.5°C) absence of fever was defined as measured body temperature below 37.5°C. Only at enrolment the absence of fever with antipyretics taken within the last 24 hours was defined as fever (enrolment criterion). The Bernoulli model was used for calculating the confidence intervals $1.96\times \surd (\frac{p\times (1-p)}{n})$.

## Results

### Desk-based analysis of dengue algorithms incorporated into IMCI guidelines

Five country guidelines were included in the desk review (Myanmar [2010], Philippines [2010], Vietnam [2015], Indonesia [2021], Cambodia [2011]). In all five countries, health workers apply the IMCI guidelines to sick children aged 2 months to 5 years according to the WHO standard IMCI algorithm by checking for five general danger signs: inability to drink or breastfeed, vomiting, history of convulsions during the current illness, lethargy or unconsciousness and convulsions.

**Fig 1:**
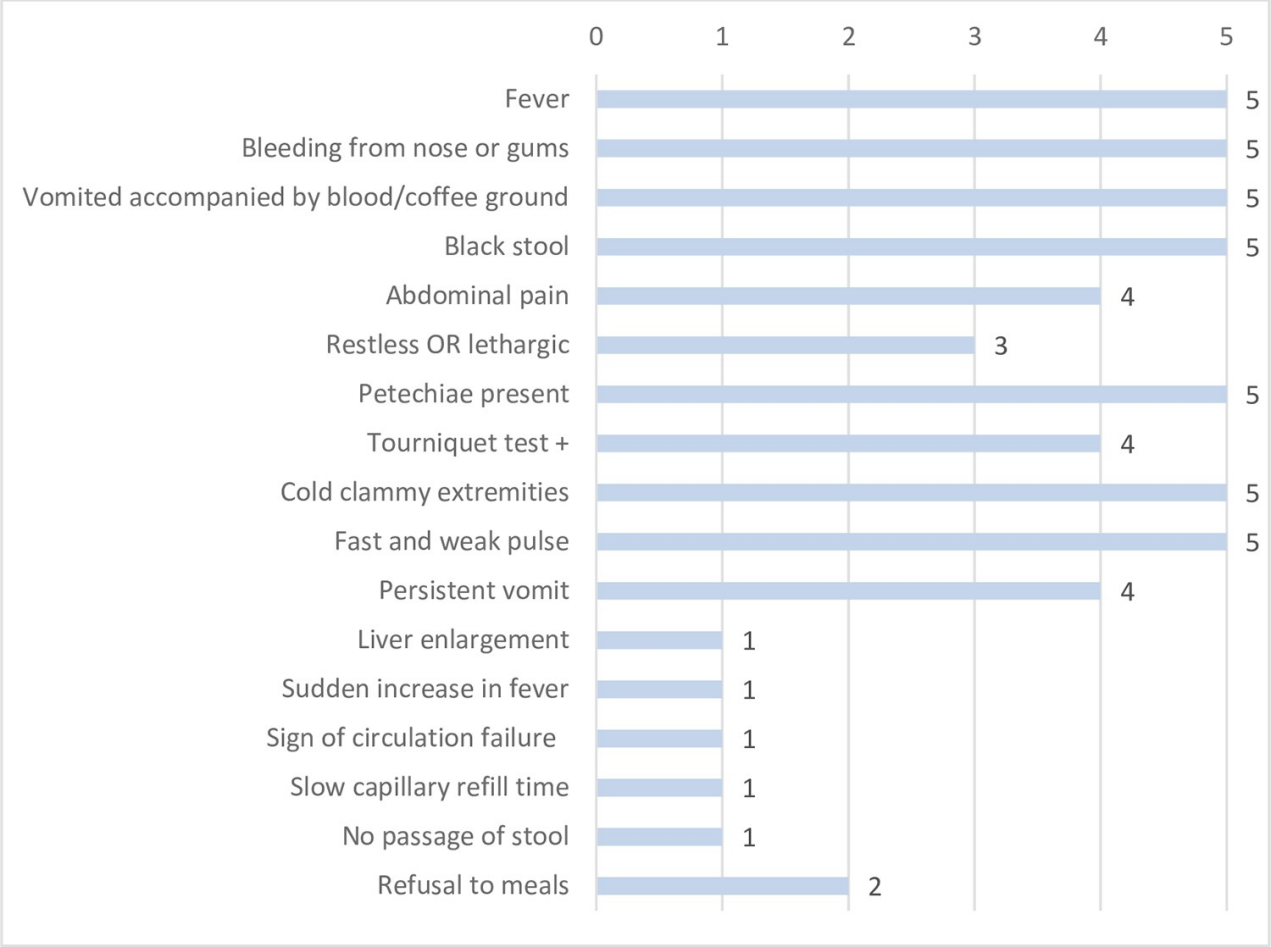
Frequency of signs and symptoms for assessment of dengue in five Southeast Asian Integrated Management of Childhood Illness (IMCI) adaptations (for children 2 month to 5 years of age).

The dengue algorithms for all five countries are integrated into the fever module for sick children aged 2 months to 5 years. Children are assessed using this module if they fulfil the WHO standard IMCI definition of “history of fever or presence of fever by palpation or measured temperature”. Only Indonesia included ‘sharp increase of fever’, and no country included ‘defervescence’ in their algorithm. Apart from fever, all countries included bleeding from the nose or gums, vomiting, black stool, petechiae and signs of shock (cold clammy extremities, fast and weak pulse) in the assessment (Fig 1). ‘Enlarged liver’ or ‘no passage of stool’ were included in the Myanmar guideline only, while ‘refusal to meals’ was explicitly named in the Cambodian and Myanmar guidelines. Once the assessment of signs and symptoms is complete, the child is classified according to severity of the illness. The classification of clinically suspected dengue used in the IMCI guidelines, varied considerably across the algorithms of the five countries. Vietnam used four different sub-categories to distinguish between dengue severity, whereas the Philippines distinguished between just two severity grades. Myanmar, Cambodia, and Indonesia distinguished between three severity grades (Table 1).

**Table 1:** Classification of clinically-suspected dengue in five Southeast Asian Integrated Management of Childhood Illness (IMCI) adaptations (for children 2 months to 5 years of age).

Country	Classification scheme
**Vietnam**	1. DF unlikely 2. DF possible 3. Severe DHF 4. DHSS
**Myanmar**	5. DF unlikely 6. DF possible 7. DHF
**Philippines**	1. DHF unlikely 2. Severe DF
**Indonesia**	1. DF unlikely 2. DF possible 3. DHF
**Cambodia**	1. DF unlikely 2. DF possible 3. DHF

The signs for requiring urgent referral to hospital in dengue algorithms were grouped into: shock, altered sensorium, bleeding tendency, and vomiting. We compared these signs across the five dengue algorithms in Table 2. All of the IMCI dengue guidelines included signs of shock (slow capillary refill time, fast and weak pulse, and cognitive variations) as indicators for urgent referral. Fluid accumulation or vascular leakage was not mentioned in any of the IMCI guidelines analysed. The guidelines from the Philippines and Myanmar did not mention explicitly cognitive impairments such as ‘abnormally sleepy’ or ‘lethargic’. Signs of shock, bleeding tendency, and vomiting were mentioned in all five guidelines.

**Table 2:** Signs requiring urgent referral of clinically-suspected dengue in five Southeast Asian Integrated Management of Childhood Illness (IMCI) adaptations (for children 2 months to 5 years of age).

Major sign	Myanmar	Vietnam	Cambodia	Philippines	Indonesia
**Shock** (cold and clammy extremities, prolonged capillary refill time, cold extremities, weak pulse)	X	X	X	X	X
**Altered sensorium** (drowsy, lethargic, difficult to wake, abnormally sleepy)	X	X	X		
**Bleeding tendency** (hematemesis, melena, bleeding from the nose, gums, gastrointestinal, petechiae, ecchymosis, back stool)	X	X	X	X	X
**Vomiting**	X	X	X	X	X
**Other**	Tourniquet test positive		Poor appetite, abdominal pain	Tourniquet test positive	

### Expert survey

The brief questionnaire was sent to 27 participants and responses were obtained from 13 of them (48%) who represented 10 different countries. The participants were from Vietnam, Cambodia, Thailand, Myanmar, Indonesia, Laos, Singapore, the Philippines, China, and Malaysia. Most of the interviewees (84%) responded that their country had a stand-alone clinical guideline for dengue, which was not integrated into the IMCI approach. The experts were in favour of an integration on dengue into the fever section of IMCI guidelines. They also welcomed an extension of the age range for the IMCI algorithm beyond 5y of age.

Another focus of the survey was the best timing for laboratory testing (full blood count) and of indicators for when a child can safely be monitored at home. The experts recommended a full blood count for children with suspected dengue during the first three days of illness, mostly between day two (38%) and day three (46%). None of the experts recommended testing (full blood count) later than day three (Table 3), assuming that the patient is getting better. Regarding monitoring at home or discharge from hospital, most respondents (92%) suggested a ‘holistic view’ of indicators based on clinical observations only, without a full blood count or other laboratory investigations necessarily being carried out. Four broad categories could be formulated based on the responses: being clinically well including having a good appetite, drinking, and urinating adequately and with stable vital signs (46%); having no warning signs according to WHO 2009 guidelines (38%); being afebrile for between 12–24 hours (8%), after 48 hours (8%) and normal platelet count (8%).

**Table 3:** Summary of results from a survey of experts on dengue algorithms.

Questions			
**Representing country**	China, Vietnam, Thailand, Myanmar, Cambodia, Indonesia, Lao, Singapore, Philippines, Malaysia		
**Is there currently a stand-alone guideline regarding dengue fever in your country?**	84% Stand-alone	8% Integrated in IMCI guideline	8% Integrated in other febrile illness guideline
**At which day of illness would you recommend or consider testing for Dengue?**	15% on Day 1	39% on Day 2	46% on Day 3
**Would you recommend integrating Dengue into the fever section of the IMCI?**	62% Yes	23% No	15% already integrated in IMCI or other febrile illness guideline

### Likelihood of dengue stratified by day of illness or number of consecutive fever days

**Fig 2:**
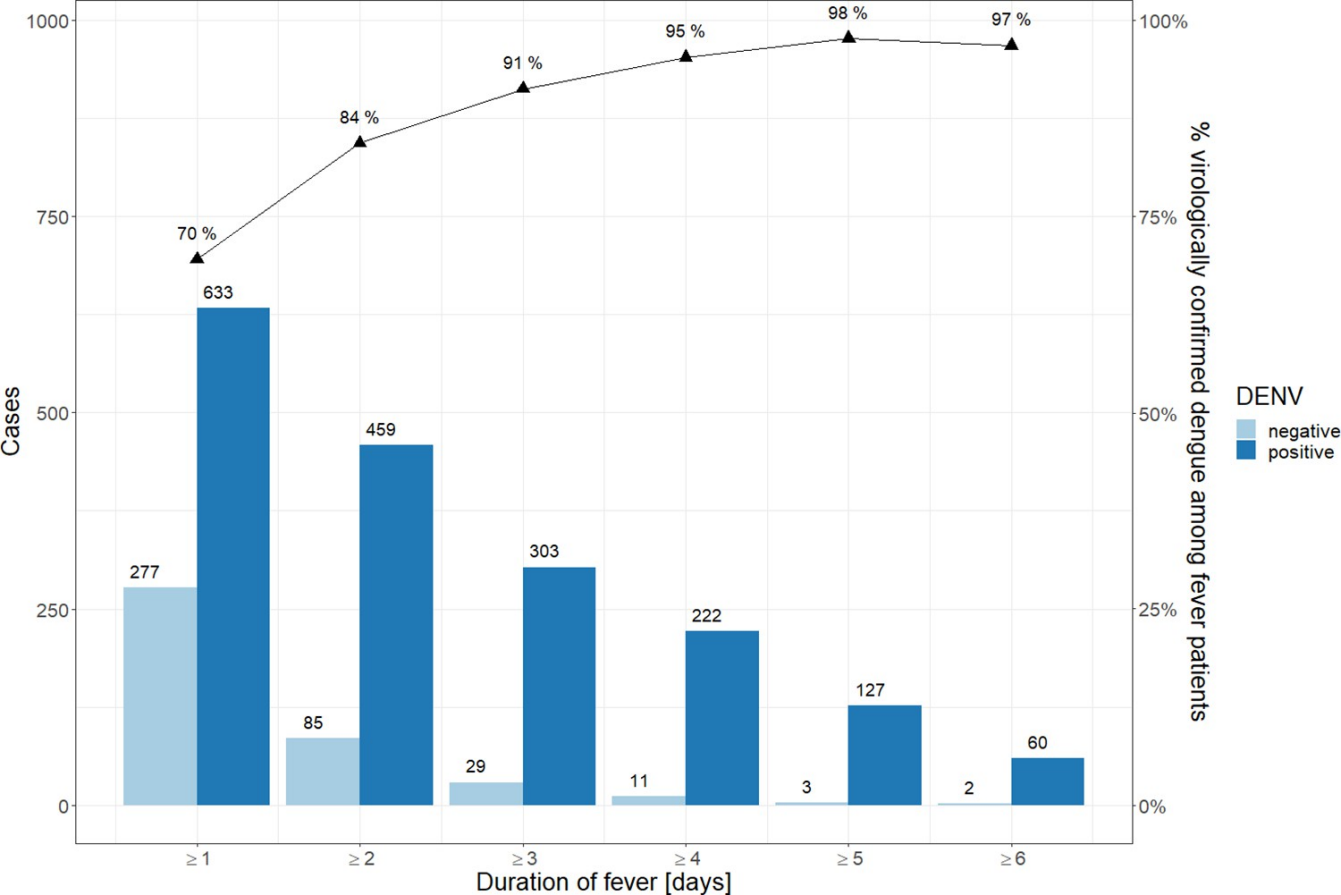
The number of children (N = 910) in the IDAMS study with positive and negative dengue diagnosis among those with consecutive fever, by day of illness. (The numbers of children per day of illness are overlapping, thus children with two, three, four etc. days of fever are also included in the first fever day).

We analysed the results from 910 children in the IDAMS study. On enrolment, all children had either documented fever or a history of fever within the last 24h (those who had taken antipyretics). The number of children with consecutive fever after two, three, four, five and six days was 544 (59.8%), 332 (36.4%), 233 (25.6%), 130 (14.2%) and 62 (6.8%). Over time, the likelihood of virologically confirmed dengue increased from 70% (633/910; 95% CI 67–73%) after at least one day of fever to 98% (127/130; 95% CI 95.6–100%) and 97% (60/62; 95% CI 92.8–100%) after five and six days of consecutive fever, respectively (Fig 2). One may note that the numbers of children per day of illness are overlapping, thus children with two, three, four, etc. days of fever are also included in the first fever day.

**Fig 3:**
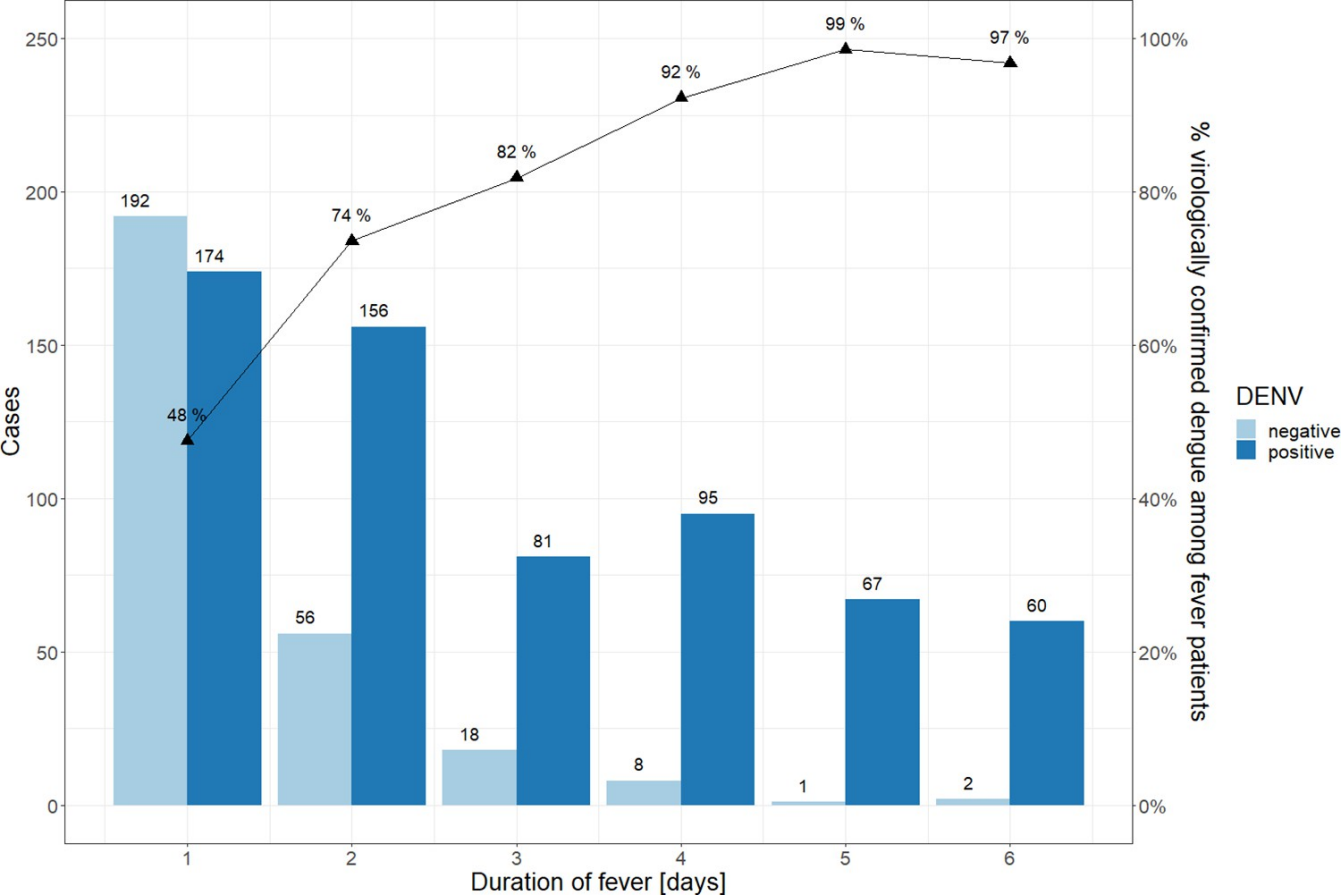
The number of children (N = 910) in the IDAMS study with positive and negative dengue diagnosis among discrete non-overlapping sub-cohorts with exact number of persisting fever days.

When we look at the results stratified by discrete non-overlapping sub-cohorts, i.e. children classified as having 1, 2, 3 etc. consecutive days of fever (Fig 3), we see the same general pattern, but with slightly lower figures. It is important to mention, however, that 174 with virologically confirmed dengue out of 910 children with fever (19.1%, 95% CI 16.6–21.7%) experienced fever for only one day and 156 with virologically confirmed dengue out of 910 children with fever (17,1, 95% CI 14.7–19.6%) experienced fever for two days. These children (330 out of 910) with a short duration of fever (1–2 days) be missed for the dengue diagnosis if testing was only carried out after three days of consecutive fever. On the other hand, none of the children with the maximum duration of three days consecutive fever had a severe course of disease in the IDAMS study.

## Discussion

We found considerable variation in the signs and symptoms sections of the five Southeast-Asian IMCI dengue algorithms, as well as in the classification schemes and the indications for urgent referral. Some signs, such as tourniquet test results and the presence of petechiae, were included in the assessment despite evidence that these signs poorly differentiate between dengue and other febrile illnesses [24–26]. Regarding the indications for urgent referral, for ease of use by first-line healthcare workers, the general signs of severe dengue included in WHO 2009 dengue guidelines were presented as more specific clinical manifestations such as cold clammy extremities, fast and weak pulse, sign of circulatory failure and slow capillary refill time, but these were found in some but not all guidelines.

Because of the nature of the IMCI focusing on the current presentation rather than the course of illness over time, the different phases of dengue (febrile, critical, recovery) and the clinical evolution were not mentioned in the dengue algorithms. While this is understandable, it may be important to put clinical signs and symptoms of dengue in the context of the expected time course. Although patients may present for care at different phases of their dengue illness (including during the critical phase), it may be important to distinguish between i) signs and symptoms for screening or diagnosis aiming at identifying dengue versus other febrile illness, and ii) warning signs ideally applied for patients with confirmed dengue disease which have prognostic value for progression to severe dengue. It is important to detect children who present with signs of severe disease and immediately refer them to hospital. However, these signs of severe disease do not necessarily serve a diagnostic purpose distinguishing between dengue and other febrile illness.

In the various dengue algorithms, warning signs for severe disease (e.g., fast, and rapid pulse, cold extremities, vomited accompanied by blood) are used in the diagnostic assessment as well as serving as indicators for referral. Importantly, some of these signs indicate the presence of severe disease rather than serving as predictors of impending disease severity. Warning signs are a well-established prognostic tool [22] for progression to severe disease, but within dengue confirmed patients. This is supported by evidence which shows the low accuracy of models that distinguish between other febrile illness and dengue based on clinical signs and symptoms alone [11,12]—or even including basic laboratory parameters such as platelet count and white blood cell count, which failed to consistently rule out dengue [27].

Conversely, this also highlights the need for point-of-care diagnostic assays for dengue in primary health care settings–as it has become standard practice for malaria diagnostic testing. In fact, the recommendation for malaria rapid diagnostic testing has been put forward from WHO and has been integrated in malaria guidelines, but not formally adapted in IMCI [28].

Regarding the optimal timing of laboratory testing, the experts recommended a full blood count on day three of consecutive fever as this approach is more conservative with regard to the cost of testing, especially in resource-limited settings. Using data from the IDAMS study, we could show that the likelihood of dengue diagnosis (by PCR/NS1) increases substantially over time. However, our results also showed that even in children with only one day of (undifferentiated) fever, around 70% were confirmed as dengue and would be missed for diagnosis or diagnosed with a delay if laboratory testing was only recommended after 3 days of consecutive fever. A sub cohort of 174 children (out of 910) (Fig 3) only had fever for one day. None of these children progressed to severe disease, but it remains to be determined if all of these children had a mild course of disease or if some of these children would still benefit from diagnosis and medical monitoring. The cost-benefit ratio of laboratory testing in settings with limited resources needs to be considered while evaluating the probability of a positive result with increasing duration of fever.

Although the majority of experts reported that dengue is assessed using stand-alone guidelines in their respective country within Southeast Asia, there was definite interest in the integration of dengue-specific content into IMCI. An integrated dengue algorithm is of importance because dengue is difficult to distinguish from other acute febrile illnesses [11,29].

In addition, the current IMCI guidelines are limited to children under five years of age while older children or adolescents represent a significant (and potentially increasing) proportion of the dengue burden. For example, dengue incidence peaked at the age of seven in a cohort in Nicaragua [30], researchers from Indonesia reported an increased risk of dengue infection until fourteen years of age [31], and a study from Thailand (from 1999) reported a peak between 5–9 years [32,33]. For Thailand, the median age of the population as a whole has been described as the driver of the force of infection, causing a shift in the age distribution of first or severe dengue infections [34].

Unsurprisingly, many experts recommend an extension of the age range of IMCI up to 16 years of age so that an integrated dengue algorithm could be applied. However, this would also require adjustments to the other components of the IMCI, which is currently mostly relevant to young children. Potentially, a partial extension of the age range could also be discussed, starting with key clinical syndromes (i.e., undifferentiated acute febrile illness) and evaluating the likelihood of dengue versus other causes based on age, geography, season, and the known presence of major infectious diseases in the region (such as malaria).

The limitations of the study include the fact that the IDAMS dataset consists of children between 5 and 15 years of age, even though IMCI targets children under the age of 5 years. Thus, the results presented on the probability of dengue by consecutive fever days are based on the age range on 5–15 years. The desk-based analysis of guidelines was conducted on the majority on Southeast-Asian countries, but some countries were not represented.

In conclusion, a standardised and validated dengue algorithm, integrated into IMCI, is desirable and would be useful for dengue-endemic Southeast Asian countries. This would ideally be combined with an extension of the age range for IMCI to include adolescents. We also provide insights regarding the optimal timing of dengue laboratory testing (via full blood count) according to the day of illness for children 5-15y. Our data shows that a considerable proportion of children would go untested if only children with three days of consecutive fever are recommended for laboratory testing. For diseases such as dengue where there is a large clinical overlap with other acute febrile illnesses, low-priced point-of-care (POC) diagnostic tests need to be made available for primary health care settings in high transmission areas [35]. In the meantime, as these POC tests are not available in the periphery, the incorporation of simple laboratory markers (complete blood count) into clinical algorithms may be beneficial for the improvement of primary health care and the timely detection of potential dengue cases.

## Supporting information

S1 Appendix Expert survey on IMCI and dengue. (DOCX)
