# Supplementary material for: Dengue algorithms integrated into the IMCI guidelines: An updated assessment in five Southeast-Asian countries
Source: PLoS Negl Trop Dis. 2022 Oct 11;16(10):e0010832. doi: 10.1371/journal.pntd.0010832 (PMC9586355; doi:10.1371/journal.pntd.0010832)
Supplement: S1 Appendix — (DOCX) [file pntd.0010832.s001.docx]

**S1 Appendix. Expert survey on IMCI and dengue.**

1. For which country or institution (WHO collaboration Center or not) are you responding?

2. Which WHO Region are you part of?

3. Is there currently a stand-alone guideline regarding dengue fever in your country – or an integrated guideline (dengue integrated in other febrile illness guideline or in an IMCI guideline)? (stand-alone, integrated in IMCI, integrated in other febrile illness guideline)

4. If Dengue is included in IMCI, when was it included?

5. If a child is coming with acute febrile illness, at which day of illness would you recommend or consider testing for Dengue? (day 1, day2, day3, day4, >day4)

6. Which are indicators when a child can safely be sent home (with or without full blood count being carried out)?

7. Would you recommend to integrate Dengue into the fever section of the IMCI? (Y/N/NA [already integrated])

8. Which age range / age extension in case of IMCI would you consider best?
